# Frameworks for health systems performance assessment: how comprehensive is Ghana’s holistic assessment tool?

**DOI:** 10.1186/s41256-020-00139-2

**Published:** 2020-03-09

**Authors:** Emmanuel Kumah, Samuel E. Ankomah, Adam Fusheini, Emmanuel Kusi Sarpong, Eric Anyimadu, Ato Quist, Brian Koomson

**Affiliations:** 1 Policy, Planning, Monitoring & Evaluation Unit, Komfo Anokye Teaching Hospital, Kumasi, Ghana; 2 Department of Preventive and Social Medicine, Dunedin School of Medicine, University of Otago, Dunedin, New Zealand; 3 Center for Health Literacy and Rural Health Promotion, P.O. Box GP1563, Accra, Ghana; 4 Biostatistics Unit, Komfo Anokye Teaching Hospital, Kumasi, Ghana

**Keywords:** Health systems, Performance assessment framework, Health indicators, Trend assessment, Developing country, Health system in Ghana

## Abstract

**Background:**

Measuring the performance of a health system is an essential requirement in creating systems that generate efficient, equitable, patient-focused, accessible and sustainable results. A fundamental requirement for a performance measurement system is the development of an assessment framework within which specific performance measures could be defined and applied regularly. This paper examines the comprehensiveness of Ghana’s health system assessment framework called the Holistic Assessment Tool in relation to some of the internationally recognized frameworks. The paper also analyzes trends in the performance of the health system to understand whether or not an improvement has been recorded following the adoption and implementation of the Holistic Assessment Tool.

**Methods:**

Mainly secondary data were used in this analysis**.** Searches were conducted on Google Scholar, PubMed, Scopus and Science Direct between May and July, 2019 for published documents on health system performance assessment. We also obtained unpublished documents from Ghana’s Ministry of Health, Ghana Health Service website, and Ghana Statistical Service database. Descriptive statistics were used to examine trends in the performance of the Ghanaian health system.

**Results:**

While the tool provides a national framework for evaluating the performance of the Ghana Health system in several domains, the Holistic Assessment Tool does not cover key health system domains such as information systems for health, access to essential medicines, and patient-centeredness. Also, the scope of the assessment program seems limited to the evaluation of the Ministry of Health’s annual plans, programs and projects. However, the health system has recorded improvements in population health indicators, such as life expectancy at birth, infant mortality, under-5 mortality, HIV prevalence and disease burden (in terms of disability adjusted life years).

**Conclusions:**

The Holistic Assessment Tool is a useful framework, but needs further refinement, both in scope and in conceptual robustness. Future studies should consider exploring factors influencing performance of the Ghanaian health system. Such information will help in strategizing for better and more improvements.

## Introduction

Health system performance assessment (HSPA) has become a central instrument in governing modern health systems. It provides a useful and rich source of evidence for policy makers in decision making regarding priority setting and resource allocation in areas of more need. HSPA also ensures tracking the progress of key strategic national health goals of specific indicators. This is especially crucial as countries make efforts towards attaining the Post-2015 Development Agenda or the Sustainable Development Goals (SDGs), particularly target 3.8 of Goal 3 (Universal Health Coverage) [1], which is at the center of current efforts of strengthening health systems [2]. By this, HSPA provides a powerful influence on policy [3]. Thus, over the last few decades, there have been several efforts towards developing comprehensive health system performance assessment frameworks that consider the distinctiveness of particular health systems, including the variety of stakeholders with different perspectives on health system performance and the various determinants of health [4, 5]. Frameworks developed largely in the context of high-income countries (HICs) have dominated these efforts [5–8]. In this regard, questions remain as to what constitutes a comprehensive health system performance assessment framework, especially, for developing and low-income countries in view of the consideration given to the peculiarities of health systems [5]. It is against this background that we adopt the definition by Tashobya et al. in this study. In their view, a comprehensive health system performance assessment framework is‘a conceptually structured way of measuring the efforts of a complex and dynamic entity; with multiple actors working in various dimensions; whose main purpose is the improvement of people’s health; the analysis of such findings; and the application of the results to decision-making’ [5]. The definition is an integration of earlier works by Sicotte and colleagues [9]; Parsons [10], and Quinn and Rohrbaugh [11]. Here, the concept of health system/healthcare organization performance is defined by the healthcare organization’s ability to maintain a dynamic equilibrium among the major dimensions of the system, namely: goal orientation; interacting with the environment; production; and maintaining internal values and norms [5, 10, 11]. This incorporates elements of both high and low income countries since Tashobya and colleague’s work focused on Uganda but built upon works in high income countries.

Measuring the performance of a health system is an essential requirement in creating systems that are resilient, responsive, efficient, equitable, patient-focused, accessible and sustainable [3]. Performance assessment also helps in understanding relationships between the performance of health system building blocks and their associated outcome indicators [3, 12]. Yet, it has been reported in the literature that many LICs are still confronted with questions of how health system stakeholders can determine if the health system is (not) performing, optimally, and what reasons account for the level of performance. This is in addition to tool(s) governments can employ to perform their stewardship role [5]. These questions underlining the measuring of health systems performance emphasize the importance of adopting an appropriate tool. According to Tashobya and colleagues, one such tool is health system performance assessment framework, the focus of this paper and, which can help answer these questions, and support evidence-based decision-making [5]. They are particularly useful in LICs given the markedly limited resources versus the huge needs but generally important in all circumstances [5].

The World Health Organization (WHO) defines HSPA as “a country-specific process of monitoring, evaluating, communicating and reviewing the achievement of high-level health system goals based on health system strategies” [13], while a health system is defined as consisting of “all organizations, people and actions whose primary objective is to promote, restore or maintain health” [12, 14]. By this, a health system is not only made up of many parts, but is also complex and comprises a number of components, including health financing, health workforce, health facilities, health therapeutics, educational and research institutions, and health care users [14, 15].

In the literature, HSPA has five main objectives. These objectives are interrelated and interdependent. They are often to set out the goals and priorities for a health system and to act as a focus for policymaking and coordinating actions within the health system. They also seek to measure progress towards the achievement of goals; to act as a basis for comparison with other health systems; and, last but not least, to promote transparency and accountability to citizens and stakeholders regarding how resources have been used [3].

A fundamental requirement for a performance measurement system is the development of an assessment framework within which exists mechanisms to define and apply regularly specific performance measures [7]. HSPA framework is a conceptual model depicting the interrelationships between the different domains and goals of a health system [16]. It gives structure and formality to the performance assessment of the various components of the health system. A HSPA framework groups health system domains under inputs, processes, outputs, outcomes and impacts, thus outlining the journey of a health system in providing and improving health. A HSPA framework also describes methods of indicator selection, sources of data used for the chosen indicators, data analysis and how results are communicated to stakeholders [17].

In 1999, WHO devised the first framework to conceptualize and assess health system performance [18]. Subsequently, the *World Health Report 2000*, considered the first attempt to comprehensively assess the performance of health systems in WHO member states, was published. Since the publication of the *World Health Report 2000*, many other international organizations such as the Organization for Economic Cooperation and Development (OECD), European Observatory on Health Systems, the European Expert Group on HSPA, the United States Agency for International Development (USAID), the Commonwealth Fund and the World Bank have undertaken several HSPA initiatives. For instance, the OECD launched its Health Care Quality Indicators (HCQI) project in 2001 and since 2007, results of the HCQI project have routinely contributed to international comparison of health systems performance [19].

An increasing number of countries have developed country-specific HSPA frameworks for periodic health systems performance assessment. The USA, for example, through the Commonwealth Fund has the National Scorecard, which assesses performance of the country’s health system in the domains of health outcomes, life expectancy, quality, access, efficiency and equity [20]. In Belgium, the Belgian Care Knowledge Center (KCE), Sciensano and the National Institute for Health and Disability Insurance jointly conduct a HSPA every three to four years, using a set of 80 indicators. England has made considerable investments in assessing the performance of the National Health Service (NHS) through monitoring a large set of indicators with targets [21]. In the Netherlands, the Dutch Health Care performance report is published bi-annually, focusing on quality, access and costs [22]. In Africa, the Health Systems Trust of South Africa publishes its district health barometer, which monitors about 20 set of indicators. The Ugandan Ministry of Health has been producing an annual health system performance report since 2011, using league table analysis introduced in 2003 to compare performance among districts and determine ‘good’ and ‘poor’ performers, and the reasons why [5]. In Ghana, there are a number of sources for measuring the health system’s performance. These include the Multiple Indicator Cluster Surveys (MICS), the Annual Health Sector in Ghana Facts and Figures publications, the Demographic and Health Surveys, but the most comprehensive yet is the Holistic Assessment Tool. It is clear that while most of the research on HSPA has been carried out in HICs; only in the recent past have a few HSPA frameworks been developed in LICs. However, experiences in LICs tend not to be explicitly documented, and few have been studied [5, 23–26]. This underscores the significance of this study as it seeks to contribute to and extend the extant literature.

Ghana’s Ministry of Health (MOH) adopted the Holistic Assessment Tool in 2008 and has since 2012 been using it to conduct annual assessment of the performance of the health sector [27]. However, since its adoption, not much has been done to assess the framework in terms of its comprehensiveness, adequacy and the extent to which the assessment program is making substantial impact on the health system of Ghana. The purpose of this paper, therefore, is to examine this Holistic Assessment Tool in relation to the literature on health system performance assessment frameworks in terms of its comprehensiveness. The objectives of the Holistic Assessment Tool are: to provide a framework for assessing the health sector comprehensively and holistically; to provide a brief, but well-informed, balanced and transparent assessment of the sector’s performance and factors that likely influenced this performance. Above all, the holistic assessment should result in recommendations of corrective measures when performance is less than anticipated through facilitating and structuring dialogue between all development partners, other key stakeholders and the government of Ghana at sector level [27].

In relation to the objectives, we addressed two main questions in this study.

1. How comprehensive is the Ghana’s Holistic Assessment Tool in relation to some of the frameworks mentioned earlier?
2. Has there been any visible improvement in the performance of the Ghanaian health system following the adoption of the Holistic Assessment Tool?

The paper is structured as follows: First, we provide a description of the methods used to conduct the study. The second section is a presentation of results under two main headings: analysis of the Holistic Assessment Tool and trends in the performance of the Ghanaian health system. The final section offers a discussion and conclusion, as well as outlining recommendations for policy, practice and research. To help readers appreciate the health system being analyzed, we have provided a brief description of the health system in Ghana in Additional file 1.

## Methods

### Study design

Data informing this study are mainly secondary in nature in the form of document analysis. The documents analysis consisted of publicly available documents. These included published peer-reviewed literature, government policy documents, legislations, Ghana Ministry of Health, Ghana Health Service, and Ghana Statistical Service websites and databases respectively. Annual reports and other grey literature formed part of the document analysis as the primary data collection method. The document analysis provided background information into the various health system assessment frameworks against which the comprehensiveness and adequacy of the Ghana’s Holistic Assessment Tool could be evaluated. Using the Holistic Assessment Tool, we then did a brief trend analysis based on some of the key indicators captured in the tool to determine whether an improvement in the performance of the Ghanaian health system could be observed following the adoption of the Holistic Assessment program.

### Document analysis

Documents examined were publicly available and obtained from both published and unpublished sources as indicated above. They included the MOH policies, plans and health sector performance assessment reports from 2012 to 2018 as the tool became mainstream in the assessment of the health sector from 2012 onwards. The others were the Ghana Health Service Annual Reports and other publications such as “the Health Sector in Ghana: Facts and Figures”, the Ghana Statistical Service databases, and documents from the World Bank home page. Four electronic databases, namely Google Scholar, PubMed, Scopus and Science Direct were searched in a non-systematic form for published documents on health systems performance assessment. The search, which focused on only English language articles published within the last 29 years (i.e. from 1990 to 2019), was conducted between May and July, 2019. Keywords used in the literature search were “health system performance assessment”, “health system performance assessment framework”, and “performance assessment indicators”.

### Data extraction and analysis procedure

#### Published documents on health system performance assessment framework

Five main HSPA frameworks, namely the WHO Building Blocks, the U.S. Agency for International Development (USAID) framework, the Control Knobs Framework, the Systems Thinking Framework, and the OECD Health Care Quality Indicators were examined and compared with the Holistic Assessment Tool. Four members of the research team, working independently, extracted information such as author’s name, date of publication and a description of the domains and indicators of the selected frameworks. Differences were discussed until a consensus was reached. To ensure data consistency and accuracy, a fifth member verified all the extracted information against each of the selected frameworks.

#### Trend analysis

Data for the analysis were obtained from MOH Holistic Assessment Reports (2014–2018), the World Bank (World Development Indicators), National Health Accounts and Demographic and Health Survey Reports. The needed information from these sources was extracted into an Excel (Microsoft Corporation, USA) database and assessed for accuracy and consistency. Time trends of the raw data extracted were then performed using 2012 as the base year. Descriptive statistics (mainly percentages and averages) were used to examine changes in performance of the selected indicators over the 7-yer period (2012–2018). We hypothesized that systematic and continuous assessment of the performance of the health system of Ghana will lead to improvements. The analysis covers changes in health status indicators (such as life expectancy, infant mortality, under-5 mortality, HIV prevalence and disease burden, among others), health workforce, health services utilization (in terms of outpatient attendance) and health spending. Indicator selection for the assessment was based on two main criteria: being a common indicator for assessing the performance of health systems in low and middle-income countries; and data availability. We do not claim this to be an exhaustive evaluation of the health system of Ghana. Nonetheless, the assessment covers some key domains of the health system.

## Results

### The holistic assessment tool: a framework for assessing the performance of the Ghanaian health system

The Holistic Assessment Tool was developed during the Ministry’s 2007–2011 Program of Work (POW) to provide comprehensive assessments of the health sector performance. The assessment is also to determine progress towards the achievement of the health sector objectives, and to serve as a feedback mechanism to development partners and other stakeholders in the sector. Further, the assessment is a way of accounting to the Ghanaian population regarding how the sector’s resources have been utilized [28].

The framework has 54 set of indicators clustered under six health sector objectives. These include bridging the equity gaps in geographical access to health services; ensuring sustainable financing for health care delivery and financial protection for the poor and improving efficiency in governance and management of the health system. The rest are improving quality of health services delivery including mental health services; enhancing national capacity for the attainment of the health related Sustainable Development Goals (SDGs) and sustaining the gains; and intensifying prevention and control of non-communicable and other communicable diseases [29].

Table 1 provides information on the 54 indicators classified under the six health sector objectives.

**Table 1:** Indicators under the Ghana’s Holistic Assessment Tool

**Objective 1: Bridge the equity gaps in geographical access to health services**	**Objective 4: Improve quality of health services delivery including mental health services**
1. Proportion of functional ambulance service centres	1. Institutional all-cause mortality
2. Proportion of functional CHPS zones	2. Proportion of regional and district public hospitals offering Traditional medicine practice
3. Per capita OPD attendance	3. Proportion of public hospitals offering mental health service
4. Equity poverty: Uder-5 Mortality Rate	4. Institutional Malaria Under 5 case Fatality Rate
5. Equity geography: Supervised deliveries	5. Surgical site infection rate
6. Equity geography: Doctor to population	6. Percentage of public hospitals with trained emergency team
7. Equity geography: Nurse to population	**Objective 5: Enhance national capacity for the attainment of the health related SDGs and sustain the gains**
8. Equity gender: Female/male NHIS active membership	1. Unmet need for contraception
**Objective 2: Ensure sustainable financing for health care delivery and financial protection for the poor**	2. Couple Year Protection(CYP), All sources incl. The private sector
1. Proportion of total MTEF allocation to health	3. Infant Mortality Rate
2. Per capita expenditure on health (USD)	4. Institutional Neonatal Mortality Rate
3. Budget execution rate (Goods and services as proxy)	5. Neonatal Mortality Rate
4. Proportion of population with active NHIS membership	6. Under-5 Mortality Rate
5. Proportion of NHIS members in exempt categories	7. Maternal Mortality Ratio
6. Proportion of population covered by NHIS as indigents	8. Institutional Maternal Mortality Ratio
7. NHIS Expenditure over Receipts	9. HIV prevalence rate
8. Equity poverty: NHIS members	
**Objective 3: Improve efficiency in governance and management of the health system**	10. Proportion of infected pregnant women who received ARVs for PMTCT
1. Doctor: Population ratio	11. Proportion of babies born to HIV mothers being HIV negative(refine)
2. Nurse: Population ratio including Community Health Nurses	12. Proportion of children U5 who are stunned
3. Midwife: WIFA Population ratio	13. proportion of children fully immunized (proxy Penta 3 coverage)
4. Proportion of health facilities in current registration	14. Antenatal Care Coverage 4+
5. Proportion of NHIF budget released to NHIS	15. Exclusive breast feeding for six months
6. Proportion of NHIS claims settled within 12 weeks	16. Proportion of deliveries attended by a trained health worker
7. Proportion of health budget (goods and service) allocated to research activities	17. Still birth rate
8. Proportion of government expenditure spent on goods and services	18. Postnatal care coverage for newborn babies
9. Proportion of government expenditure spent on assets	19. Proportion of children under 5 years sleeping under ITN
	20. TB treatment success rate
	**Objective 6: Intensify prevention and control of non-communicable and other communicable diseases**
	1. Non-Acute Flaccid Paralysis (AFP) Polio rate
	2. Population prevalence of hypertension
	3. Number of deaths attributable to selected cancer

Source: MOH, 2015 [18]

*MTEF* Medium-Term Expenditure Framework, *NHIF* National Health Insurance Fund, *WIFA* Women in Fertility Age, *PMTC* Prevention of Mother to Child Transmission, *ITN* Insecticide Treated Net.

Since 2012, the tool has been applied annually to assess the performance of the health sector. The analysis underlying the annual assessment is based on five key elements:

1. The health sector’s annual POW,
2. Annual performance review reports and presentations of the Ministry and its agencies,
3. Annual financial statements of the Ministry,
4. National survey reports, and
5. The Health Sector Medium Term Development Plan (HSMTDP) [27].

The assessment process involves three steps. The first step entails assessing each indicator and milestone. The second step focuses on grouping the indicators and milestone values under the health sector objectives and computing subtotals for each group. The final step is about assessing the overall sector performance by adding the scores of each of the health sector objectives. This last step in the process is assessed on a scale of 0–5 with five quintiles. A score of 4–5 or within the highest quintile means the sector is highly performing. A score within the next or second highest quintile of 3–4 implies the sector is moderately performing. A score within the middle quintile of 2–3 is interpreted to mean the sector performance is being stagnant. A score within the second lowest quintile of 1–2 shows the sector is underperforming; while a score within the lowest quintile of 0–1 implies the sector is severely underperforming [30].

The output of the assessment is a comprehensive report covering an analysis of the progress of each indicator, an assessment of each health objective, and of the overall sector performance. The report also covers the extent to which the sector’s priority activities have been implemented. At a National Annual Performance Review Meeting and Health Summit, the report is firstly presented and discussed. Suggestions and recommendations are discussed at the sector’s Business Meeting, and the report is finalized and published [30].

### Adequacy and comprehensiveness of the holistic assessment tool: a comparison with common health system frameworks

The health sector objectives and the indicators under the Holistic Assessment Tool (Table 1) could be classified into health system domains such as access and equity, financing and financial risk protection, efficiency, governance, quality (of service delivery), health status, safety, and coverage. The indicators could also be classified along input (service delivery, human resource, financing, governance etc.), throughput or intermediary output (quality, equity, safety, access, coverage), and outcome (health status) dimensions, in line with Donabedian’s structure, process and outcome model [28].

It has been asserted that the adequacy and effectiveness of a performance measure depends on data quality and the extent to which the chosen assessment framework reflects the objectives of the health system [31]. The key domains of the Holistic Assessment Tool are the health sector objectives of Ghana. Thus, the framework adequately reflects the objectives of the Ghanaian health system. However, data completeness, in terms of availability of all of the required information for the assessment, is sometimes lacking. The assessment method uses survey-based information from national survey reports to compute some of the indicators [27] such as maternal mortality ratio, neonatal mortality, infant mortality and under-5 mortality rates. National surveys are normally conducted between every two to five years. Consequently, data on these indicators are included in the assessment only when new information is available. For instance, in 2017, the Holistic Assessment Report did fail to report on certain key indicators due to the unavailability of national survey reports for that year.

One of the frameworks that has been used widely in health systems research and has become a benchmark/gold standard commonly used to describe a health system is the WHO Building Blocks [12, 32]. The framework describes a health system in terms of six components: (i) service delivery, (ii) health workforce, (iii) health information systems, (iv) access to essential medicines, (v) financing, and (vi) stewardship/governance [12]. Stewardship/governance and health information systems provide the basis for the overall policy and regulation of the other building blocks; financing and health workforce constitute the input components of the health system, whereas service delivery and medical products/technologies reflect the immediate outputs of the system [17]. Indicators published by the Ghana’s Holistic Assessment Tool could be classified as reflecting four of the WHO Building Blocks: service delivery, health workforce, financing, and governance. However, none of the 54 indicators could be related to the other two components of the Building Blocks: health information systems and access to essential medicines. According to WHO, a well-functioning information system provides the foundations for decision-making. Indicators of a country’s health information system could be grouped into two broad categories: indicators related to data generation, using core sources and methods (i.e. health surveys, civil registration, census, facility reporting and health system resources tracking); and those related to country capacities for synthesizing, analyzing and validating data [17]. Access to essential medicines is about ensuring equitable access to essential medical products, vaccines and technologies of guaranteed quality, safety, efficiency and cost-effectiveness. Recommended indicators for measuring access to essential medicines include: tracer drug availability in health facilities, and median drug price ratio for tracer drugs [17].

Building on the WHO framework, the U.S. Agency for International Development (USAID) developed the “Health Assessment Approach: A How-to Manual”. This has been widely applied to the assessment of some low and middle-income countries’ health systems, including Nigeria, Vietnam, Benin, Senegal, Angola, Lesotho, Kenya, South Sudan and Zimbabwe [33]. The USAID framework considers a health system as covering governance, health financing, human resource for health, service delivery, pharmaceutical management, and health information systems [33]. The Ghana’s HSPA, while covering four domains of the USAID framework (governance, financing, human resource and service delivery), does not cover two: pharmaceutical management and health information systems.

Furthermore, one important HSPA domain highlighted by other international health system frameworks - such as the Control Knobs Framework [34], Systems Thinking Framework [35, 36], and the OECD Health Care Quality Indicators (HCQI) Framework [6] - but not included in the Holistic Assessment Tool is patient-centeredness. WHO uses measures of patient centeredness as an indicator of a health system’s responsiveness, defined as the ability of a health system to respond to the expectation of users about non-health enhancing aspect of care [37]. Porter has suggested that the accountability of health systems for producing value should be defined around the user [37]. Proposing an alternative framework for assessing the performance of health systems in developing countries, Kruk and Freedman identified patient satisfaction (a proxy measure of patient-centeredness) as one of the measures of health system effectiveness, alongside improvement in health status, access to and quality of care [38]. A recent publication in Lancet Global Health argues that among the key dimensions on which health systems should be judged is people-centeredness, defined as systems that are easy to navigate, with short wait times and attention to users’ values and preferences [39]. Some of the common indicators used to assess people-centered health systems are: patient satisfaction, respect for patients’ dignity, patient choice, prompt attention to medical needs, and involvement of the patient or public in health system governance and accountability [3].

In summary, the Ghana’s Holistic Assessment Tool could be described as limited in scope in terms of its ability to assess the entire health sector of Ghana. Health system performance assessment encompasses measuring and analyzing how well a health system is meeting its overall goals (i.e. improved health, responsiveness to people’s expectations, social and financial protection and improved efficiency) [40], and how its performance against intermediate outputs (e.g. access, coverage, quality and safety of health services) contributes to achieving these goals [12]. Per the literature, a comprehensive HSPA covers the entire health system and not limited to specific programs, objectives or levels of care [41]. However, the Holistic Assessment Tool does not cover key health system dimensions such as information systems for health, access to essential medicines, and patient-centeredness. In addition, the scope of the assessment program seems limited to the evaluation of the health sector’s annual plans, programs and projects.

#### Performance of the health system of Ghana

**Fig. 1:**
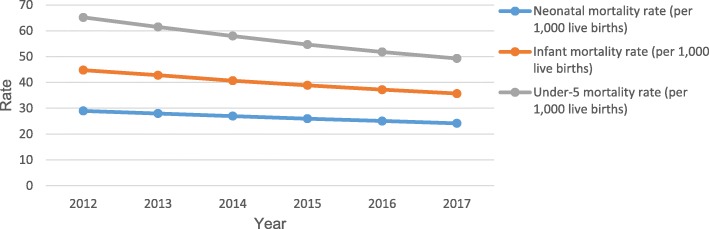
Trends in childhood mortality (Source: World Bank, World Development Indicators [42]

**Fig. 2:**
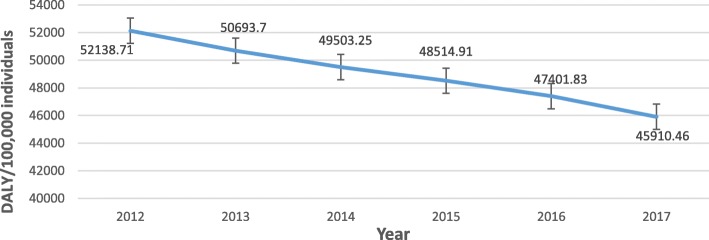
Age standardized DALY rate per 100,000 individuals from all causes (Source: Roser & Ritchie [43]

Table 2 shows trends in the selected indicators between 2012 and 2018. The country’s population health indicators have shown some improvements. Life expectancy at birth (total), increased from 61.56 years in 2012 to 63.4 years in 2018, recording an average annual increase of 0.49% (0.46–0.59%). Childhood mortality indicators have shown a consistent decline (Fig. 1). Infant mortality rate, for instance, decreased from 44.8 deaths per 1000 live births in 2012 to 35.7 deaths per 1000 live births in 2017. Under-5 mortality rate also fell from 65.2 deaths per 1000 live births in 2012 to 49.3 deaths per 1000 live births in 2017. Some improvements could also be observed in morbidity indicators. Prevalence of HIV among adults (aged 15–49) has shown an average annual decrease of 1.8% (0–5.26%), decreasing from 1.9% in 2012 to 1.7% in 2018. Malaria under-5 case fatality decreased from 0.76% in 2012 to 0.16% in 2018, with an average annual decrease of 21.9% (5.6–37.5%). To sum it, Disability-Adjusted-Life Year (DALY), considered a summary measure of population health, has shown a downward trend, falling from 52,138.7 DALYs lost per 100,000 individuals in 2012 to 45,910.5 DALYs lost per 100,000 individuals in 2017 (Fig. 2).

**Table 2:** Trends in the performance of the Ghanaian health system

Indicator	2012	2013	2014	2015	2016	2017	2018
**Health status indicators:**							
Life expectancy at birth, total (years)	61.56	61.86	62.15	62.45	62.74	63.03	63.4
Death rate, crude (per 1000 people)	8.8	8.7	8.4	8.3	8.1	8.0	–
Neonatal mortality rate (per 1000 live births)	29	28	27	26	25.1	24.2	–
Infant mortality rate (per 1000 live births)	44.8	42.8	40.7	38.9	37.2	35.7	–
Under-5 mortality rate (per 1000 live births)	65.2	61.5	58	54.7	51.8	49.3	–
Prevalence of HIV, total (% of population ages 15–49)	1.9	1.8	1.8	1.7	1.7	1.7	1.7
Malaria under-5 case fatality rate	0.76	0.69	0.54	0.51	0.32	0.2	0.16
Tuberculosis treatment success rate	84	85	87	85	86	87	86
DALY rate per 100,000 individuals	52,138.7	50,693.7	49,503.3	48,514.9	47,401.8	45,910.5	–
**Health workforce indicators:**							
Physician to population ratio	1:11,515	1;10,170	1:9043	1:8934	1:8301	1:8100	1:7196
Nurse to population ratio	1:1362	1:1084	1:959	1:865	1:834	1:799	1:839
Midwife to women in fertility age ratio	1:1611	1:1525	1:1374	1:1216	1:943	1:746	1:689
**Health financing indicators:**							
Total health expenditure as a % of GDP	5.5	6.1	5.6	6.1	4.4	–	–
Health expenditure as a % of government expenditure	7.6	9.3	10.6	7.0	6.8	6.5	–
Out-of-pocket expenditure (% of current health expenditure)	39.8	38.9	45.1	35.8	37.8	–	–
Per capita expenditure on health (US$)	89.85	111.04	81.52	82.41	67.51	–	–
**Health services utilization:**							
Outpatient services utilization per capita	1.17	1.16	1.15	1.08	1.06	0.98	1.05

*DALY* Disability Adjusted Life Years*.*

Data sources: Ministry of Health Ghana Holistic Assessment Report, 2014–2018; The World Bank; National Health Account of Ghana, 2015; Ghana Demographic and Health Survey, 2014.

Some improvements have also been made in the country’s health workforce. Physician to population ratio improved from one physician per 11,515 population in 2012 to one physician per 7196 population in 2018, however, this is not across the country. Similarly, nurse to population ratio has improved from 1:1362 population in 2012 to 1:839 population in 2018. The ratio varies in some regions of the country though. Further, midwife to women in fertility age ratio recorded an improvement from 1:1611 women in 2012 to 1:689 women in 2018 with variations across regions [44]. Population densities, supply-side behavior of some higher-level cadres towards urban employment, the perception of better urban working conditions both in terms of career development prospects and in workloads, clinical infrastructure, social life, and income explain some of these variations [45].

**Fig. 3:**
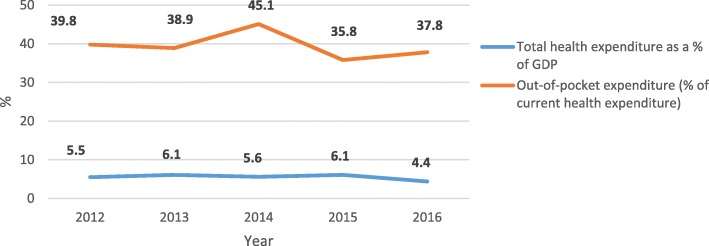
Trends in health spending indicators (Sources: *National Health Account of Ghana* [46]*; World Bank, World Development Indicators* [42]

On the contrary, health financing indicators have not shown any improvement. Trend in government spending on health as a percentage of GDP has been fluctuating (Fig. 3). Per capita expenditure on health increased in 2013 (US$111.04), but has since shown a downward trend. Trend in out-of-pocket spending as a percentage of current health expenditure has also been erratic, and between 2012 and 2016, no significant reduction has been recorded (Fig. 3). These results are an indication that the health system has a relatively weak outcome on financial protection.

**Fig. 4:**
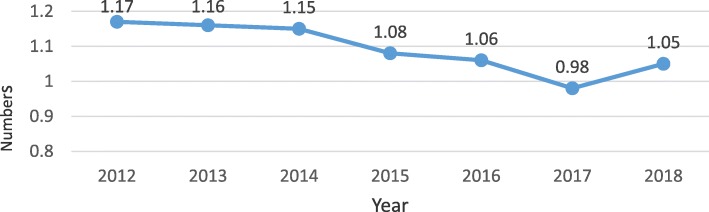
Outpatient visits per capita (Source: Ministry of Health Ghana Holistic Assessment Reports 2014, 2017 & 2018)

Performance in the number of outpatient consultations per person per year, which is considered a proxy indicator for accessibility, and utilization of health services, has not been encouraging. As indicated by Fig. 4, outpatient visits per capita showed a downward trend from 2012 to 2017, with an average annual reduction of 3.4% (0.85–7.34%), and increased marginally between 2017 and 2018. The 2018 increase (1.05) is still below the 2012 performance of 1.17.

As these are secondary analyses of documents, we assume that the adoption of the tool might have focused policy makers’ attention in making decisions regarding some of the priority areas requiring more attention, hence the improvement witnessed. In light of this, we could not concretely conclude this is the case, as further research will be required to make a definitive conclusion.

## Discussion

The development and implementation of Ghana’s Holistic Assessment Tool is an important milestone towards creating a more transparent and accountable health system, while allowing policy makers in the country the opportunity to focus on areas requiring more attention and improvement. However, our analysis has revealed that the assessment framework does not cover all of the key health system dimensions. The framework, therefore, falls short of the definition of a robust and comprehensive HSPA framework given by Papanicolas and Smith as one that embraces all of the salient components of a country’s health system [3].

Notwithstanding, it could be argued that Ghana’s HSPA tool is not the only framework that does not cover all of the key health system domains as defined by international organizations, such as WHO, the World Bank and USAID. For instance, a recent study by Fekri et al. to determine the domains and indicators used by Member States in the WHO European Region in their HSPA found that no single Member State was publishing indicators across all of the domains of the WHO 2007 Framework [40]. In practice, countries narrow the scope of their framework so the assessment could be aligned with identifiable improvement actions to ensure greater accountability. This practice has, however, resulted in many of the processes and outcomes that matter most to the public not being captured [39]. Broadening the scope, therefore, presents a more comprehensive understanding of all the factors that determine health. In the case of Ghana, the scope of the Holistic Assessment Tool needs broadening to cover indicators assessing patient-centeredness, access to essential medications and health information system.

Placing the service user at the center of healthcare delivery is an integral component of Ghana’s National Healthcare Quality Strategy (2017–2021). It is thus surprising that no single indicator under the Holistic Assessment Tool assesses patient centeredness. Presently, no national data even exists on overall user experience and satisfaction with the healthcare system. Although, some patient satisfaction surveys have been conducted, these studies have been restricted to either certain geographical locations [47] or specific types of care [48], thus limiting the generalizability of the findings to the entire country. Consequently, one key question arises - how does the country assess progress towards attaining patient centeredness in healthcare delivery as contained in its National Healthcare Quality Strategy? To answer this question, considerable attention must be placed on assessing the centeredness of the health system to the needs of the public.

One of the key features of performance measurement is that it is regular [41]. The Ghana’s HSPA which is conducted annually [27] is highly commendable. However, because information for some of the indicators is obtained from reports of national surveys, which are not conducted annually, the assessment exercise, in some of the years, is challenged by data availability. To overcome this limitation, the Ministry of Health may reconsider the frequency and timing of the assessment to ensure that the exercise is always done just after some of these national surveys. By this, assessment of the health services delivery performance could be done annually, while that of the whole health system could be done bi-annually or every three to four years as is being done in the Netherlands and Belgium respectively.

We observed a lack of description of the conceptual basis for the Holistic Assessment Tool. This information is normally necessary in understanding the content and performance dimensions of any HSPA framework [3]. For instance, the UK’s HSPA framework is based on a balanced scorecard approach; the Canadian framework is based on the population health model; while that of Australia is based on a health determinants model [23]. Adding a description of the conceptual basis for the Ghana’s HSPA framework is thus necessary.

As the main goal of a health system is to improve the health of the population it serves [39], the marginal improvement in the health status indicators we observed might be an indication that the performance of the Ghanaian health system is improving, although no improvements were recorded in the health spending indicators. It is, however, vital that the country’s policy makers focus more attention on improving outpatient services utilization. Even though it is argued that the number of outpatient visits does not measure actual utilization of services because people may make repeated visits, low rates are considered an indicative of lack of availability and poor quality of services [49]. For instance, it has been demonstrated in several countries that outpatient visit rates increase when barriers to using health services, such as reducing user fees and bringing services closer to the people are removed [50].

### Further research

Our attempt to assess the health system’s performance was limited by gaps in availability and quality of data. Reliable data for trends in many key indicators were missing. This limitation did not allow us to conduct a detailed assessment of trends in the performance of the health sector. More rigorous, countrywide studies assessing the performance of the Ghanaian health system, using some of the internationally recognized HSPA frameworks as theoretical bases, are therefore required. Also, the Holistic Assessment program might be just one of the factors accounting for the improvement in the population health indicators. Further studies are therefore needed to understand factors influencing performance of the Ghanaian health system. This information will help in strategizing for better and more improvements.

## Conclusions

Monitoring and evaluating overall health system performance is a complex and challenging task, but this is critical to ensuring better performance of the health system. The present paper principally assessed the comprehensiveness of the Ghana’s HSPA framework in relation to internationally recognized frameworks such as the WHO Building Blocks, the Control Knobs and the Systems Thinking Framework. Our analysis has revealed that the Holistic Assessment Tool, though a useful monitoring and evaluation framework, does not cover some key health system domains. Further refinement of the framework, both in scope (in terms of the indicators published) and in conceptual robustness is thus warranted.

## Supplementary information

**Additional file 1.** An overview of Ghana’s Health System [51–56].

## Data Availability

The data used in this analysis are available from the corresponding author on request.

## Abbreviations

CHAG: Christian Health Association of Ghana
CHPS: Community-Based Health Planning and Services
DALY: Disability Adjusted life year
DMHIS: District Mutual Health Insurance Scheme
GDP: Gross Domestic Product
HCQI: Health care quality indicators
HICs: High-income countries
HSMTDP: Health sector medium term development program
HSPA: Health system performance assessment
LICs: Low-income countries
MOH: Ministry of Health
NHIS: National Health Insurance Scheme
NHS: National Health Service
OECD: Organization for Economic Cooperation and Development
POW: Program of work
SDG: Sustainable development goals
USAID: United States Agency for International Development
VAT: Value added tax
WHO: World Health Organization
