# Supplementary material for: Frameworks for health systems performance assessment: how comprehensive is Ghana’s holistic assessment tool?
Source: Glob Health Res Policy. 2020 Mar 9;5:10. doi: 10.1186/s41256-020-00139-2 (PMC7061475; doi:10.1186/s41256-020-00139-2)
Supplement: Supplementary file 1 — Additional file 1. An overview of Ghana’s Health System [51–56]. [file 41256_2020_139_MOESM1_ESM.docx]

**An overview of Ghana’s Health System**

Every country has a national health system, reflecting its history, economic development and political ideology [51]. The Ghanaian health system is no exception. Meanwhile, although health systems have been classified differently, the classifications share many common concepts and distinguish three major dimensions of health systems: regulation, health services provision and financing [52]. The Ghanaian health system presented below is along these three dimensions.

Ghana’s health system is anchored on the Ministry of Health (MOH). The Ministry has the oversight responsibility of regulating the entire health sector. Its main function involves policy formulation, coordination and regulation of the stakeholders in the health sector [27]. The Ministry has about 24 agencies through which its functions are performed, in conjunction with various ministries and departments, as well as key development partners and stakeholders within the health sector. The agencies are classified under five main functions: regulatory, service delivery, financing, research and training [27] (Table 1). These agencies continue to expand in line with changing trends of the country’s population.

| **Table S1. Agencies under the Ministry of Health (MOH), Ghana** | | |  |  |
| --- | --- | --- | --- | --- |
| **Regulatory** | **Service delivery** | **Financing** | **Research** | **Training** |
| Food & Drug Authority | Ghana Health Service | National Health Insurance Authority | Center for Plant & Medicine Research | Ghana College of Physicians & Surgeons |
| Pharmacy Council | Tamale Teaching Hospital |  |  | Ghana College of Pharmacists |
| Psychology Council | Komfo Anokye Teaching Hospital |  |  | Ghana College of Nurses and Midwives |
| Nursing & Midwifery Council | Korle-Bu Teaching Hospital |  |  |  |
| Medical & Dental Council | Cape Coast Teaching Hospital |  |  |  |
| Health Facilities Regulatory Agency | Christian Health Association of Ghana |  |  |  |
| Traditional Medicine Practice Council | Ghana Association of Quasi Government Health Institutions |  |  |  |
| Allied Health Professional Council | Mental Health Authority |  |  |  |
|  | Ahmadiya Muslim Mission |  |  |  |
|  | Mortuary Services Agency |  |  |  |
|  | National Ambulance Service |  |  |  |
|  | National Blood Service |  |  |  |

**Source:** <http://www.moh.gov.gh/category/ministry-agencies/>

Service provision is organized under four main categories of delivery system: public, private-for-profit, private-not-for-profit and traditional systems. The public sector operates a multi-level healthcare delivery system. At the bottom of the hierarchy are community-based health planning and services (CHPS) zones mandated to provide basic healthcare at the community level [30]. There are also health centers at the sub-district levels providing basic curative and preventive services to populations of 20,000. In the urban areas, these health centers are upgraded to polyclinics serving populations larger than 20,000. District hospitals serve as referral centers for the health centers and provide basic and emergency healthcare to populations of 100,000 – 200,000. Next in the hierarchy are regional hospitals, providing secondary levels of specialist care and serving as referral centers for each of the political regions in the country. Top on the hierarchy are four teaching hospitals (increasing to five with the Ho Teaching hospital), with the mandate of managing complex health conditions, undertaking research and training of health professionals [53]. The health system is made up of a variety of facility types and affiliations, ranging from government-owned to privately owned facilities. Approximately 57% of health facilities are public, 33% are private and 7% are operated by the Christian Health Association of Ghana (CHAG) [54].

The health system is financed mainly from diversified sources including non-tax revenues, taxes (both direct and indirect), donor contributions and out-of-pocket payments from individuals [32]. Ghana is among the few lower middle-income countries implementing a national health insurance scheme (NHIS). The NHIS is funded from several sources, including 2.5% value added tax (VAT) levied on selected goods and services, 2.5% social security deductions from formal sector workers, government annual budgetary allocations proposed and approved by parliament to the scheme, and voluntary contributions (premiums) paid by subscribers to the scheme [55]. Other sources include investment income of the National Health Insurance Fund (NHIF), grants, donations, and gifts. The rest consists of any voluntary contribution to the NHIF and fees charged by the National Health Insurance Authority in the performance of its functions. Finally is money accruing under section 198 of the Insurance Act of 2006 [56].The National Health Insurance Act, 2012 (Act 852) permits the operation of three types of insurance schemes: the national health insurance, private mutual health insurance and private commercial insurance schemes. The benefit package of the NHIS covers outpatient and inpatient services (about 95% of common illnesses in Ghana) at accredited facilities (both for-profit and not-for-profit), as well as community based health planning services compounds. Both public and private providers are paid by the NHIA via three payment mechanisms- fee-for-service, diagnostic related groupings and capitation (Pilot). Public and some private-not-for-profit facilities (such as those affiliated with CHAG) receive funding allocations on the basis of historical budgets. Private-for-profit practitioners render services on a fee-for-service basis through out-of-pocket payments [56].
